# Nine decades of data on environmental chemical pollutant exposure in dogs: a bibliometric analysis

**DOI:** 10.1007/s11356-022-24791-y

**Published:** 2023-02-21

**Authors:** Albert Avila, Laura Prieto, Andrea Luna-Acosta

**Affiliations:** 1 Departamento de Ecología y Territorio, Facultad de Estudios Ambientales Y Rurales, Pontificia Universidad Javeriana, Transversal 4 # 42-00, Edificio 67, Piso 8, Bogotá, Colombia; 2 Departamento de Biología, Facultad de Ciencias, Pontificia Universidad Javeriana, Carrera 7 # 43-82, Bogotá, Colombia

**Keywords:** Bibliometric, Environmental chemical pollutants, Exposure, Effects, Dogs, Sentinel

## Abstract

In recent decades, a global concern associated with environmental chemical contamination has emerged as an important risk factor for the development of human diseases. Risk assessment methods based on animal approaches have shown to be very useful as early warning systems. However, questions, knowledge gaps, and limitations still need to be addressed in animals close to humans, such as dogs. The objective of this study was to analyze citation patterns, impact of publications, and most relevant authors, countries, institutional affiliations, and lines of research on environmental chemical contaminants and their relationship with dogs, in terms of exposure and biological effects. For this, a bibliometric analysis was carried out. Results revealed an increase in scientific production on this subject during the last 90 years in journals such as *Health Physics*, *Science of the Total Environment*, and *Plos One*, highlighting authors such as Muggenburg, Sonne, Boecker, and Dietz. The USA, Brazil, Germany, and the UK and universities such as California, Colorado State, and Purdue were the most relevant countries and institutional affiliations in scientific production and collaboration in relation to this topic. There is a growing interest in the development of lines of research related to heavy metals (mercury and lead mainly) and persistent organic compounds (PCBs, PBDEs, pesticides) using dogs as sentinels, as well as new sources of interest related to zoonosis and One Health. Finally, issues related to pollutants, sentinel lymph nodes, and epidemiology appear as new areas of research. These results highlight interesting current challenges and future research perspectives on dogs as sentinels for environmental chemical contamination.

## Introduction

Environmental chemical pollutants may have negative effects on living organisms and surrounding environment (Thompson and Wageh 2019). This broad group includes persistent organic pollutants (POPs), such as polychlorinated biphenyls (PCBs), polycyclic aromatic hydrocarbons (PAHs), perfluorinated compounds (PFCs), pharmaceuticals, and personal care products (PPCPs) (Aryal et al. 2020). It also includes radioactive elements and inorganic compounds such as heavy metals and nanoparticles, among others (Aryal et al. 2020; Pastorinho and Sousa 2020a, b). There is growing evidence of a considerable increase of some of these elements in recent years, both in concentration and geographical distribution (Córdoba 2006; Wasi et al. 2013; Aryal et al. 2020; Carriquiriborde 2021). Some of these compounds occur in natural sources (Jyothi 2020). However, contemporary anthropogenic activities, such as intensive industrial activities, land-use changes, mining, and fossil fuel combustion, are considered main factors that release these chemicals into ecosystems (Conti 2008; Selin 2009). Once liberated in the environment, some of these compounds are biotransformed, bioaccumulated, and/or biomagnified in exposed organisms, having a wide range of biological effects (Andreoli and Sprovieri 2017; Boisvert et al. 2019). The main routes of exposure to these contaminants in animals and humans are diet, soil, water, and air (Laks 2007; Thompson and Wageh 2019; Pastorinho and Sousa 2020a).

In recent years, there has been a great concern associated with environmental pollution as a risk factor for the development of human diseases (Chen et al. 2007; Laks 2007; Ipenza 2012; Wasi et al. 2013; Obiri et al. 2016; Schutzmeier et al. 2017; Sonne et al. 2020). Different models have been used to assess environmental chemical contaminants, in particular animals, used as sentinels of human exposure to pollutants (Abbott 2017; Pastorinho and Sousa 2020a). Dogs have very similar enzymatic and metabolic systems to humans, a physiologically compressed lifetime (Wang et al. 2020a, b), and are free of risk factors for lifestyle-related diseases (Parker et al. 2006; Abbott 2017; Fuentes et al. 2018). In addition, dogs share the same environment, responding to many toxic factors in an analogous way. Dogs can be used as a feasible and low-cost alternative when compared to large-scale studies in humans (Kucera 1988; Toyomaki et al. 2020). In addition, dogs have shorter latency periods, due to briefer lifespan (Hoffman et al. 2018). Therefore, dogs are a suitable study model and can be used as early warning systems, allowing timely public health interventions, especially in regions of the world where short-term, high health reliability and low-cost results are required (Kucera 1988; Toyomaki et al. 2020). However, there are several knowledge gaps that prevent the identification of true cause and effect relationships of contaminants in dogs.

Bibliometric analyses are quantitative statistical analysis tools frequently used in the fields of publishing and information science, to provide quantitative and objective analysis of academic literature (Aria and Cuccurullo 2017; Pranckute 2021; Benavent et al. 2017; Cooper 2015), including issues associated with environmental pollution (Zhang et al. 2017), climate change (Sweileh 2020), and One Health (Humboldt et al. 2020). The generation of bibliometric articles allows in a simple and robust way the evaluation of patterns and trends that should be considered when addressing knowledge gaps (Heinninger 2012). Under this context, the main objective of this study was to carry out a bibliometric analysis on environmental chemical pollutants and their relation to dogs, in terms of monitoring and biological effects during the last 90 years. The purpose of this study was to analyze (1) the evolution over time of the number of publications; (2) main publication sources, authors, institutional affiliations, and countries; (3) the most developed topics; and (4) the least developed topics. This was done to confirm if there is a growing interest on this research field, to highlight journals, authors, and countries that should be consulted for the development of future collaborations. This study is aimed also at being the first approximation to the identification of patterns of publications in this scientific field. This could help to objectively develop guidelines for future research studies using dogs as sentinel species.

## Materials and methods

An advanced search was carried out in “Scopus,” under the following key terms: dog* OR dogs* OR “canis familiaris” OR “canis lupus familiaris” OR canine*) and “environmental pollutant*” OR “chemical pollutant*” OR “persistent organic compound*” OR “persistent organic pollutant*” OR “inorganic pollutant*” OR “sentinel*” OR “air pollutant*” OR “water pollutant*” OR “soil pollutant*” OR “hydrocarbon pollutant*” OR “brominated compounds*” OR “heavy metal*” OR “pesticides*” OR “herbicides*” OR “microplastics*” OR “polycyclic aromatic hydrocarbons*” OR “polychlorinated biphenyls*” OR “polybrominated diphenyl ethers*” OR “pharmaceuticals*” OR “personal care products*” OR “emerging pollutants*” OR “nanomaterials*” OR “radionuclides*”). Other animals used as sentinels of environmental chemical contamination were excluded, as follows: Exclude (Exactkeyword, “Cat”) Or Exclude (Exactkeyword, “Rats”) Or Exclude (Exactkeyword, “Rat”) Or Exclude ( Exactkeyword, “Cats”) Or Exclude (Exactkeyword, “Mouse”) Or Exclude (Exactkeyword, “Mice”) Or Exclude (Exactkeyword, “Rabbits”) Or Exclude (Exactkeyword, “Cattle”) Or Exclude ( Exactkeyword, “Swine”) Or Exclude (Exactkeyword, “Rabbit”) Or Exclude (Exactkeyword, “Cat Diseases”) Or Exclude (Exactkeyword, “Fish”) Or Exclude (Exactkeyword, “Sheep”) Or Exclude (Exactkeyword, “Guinea Pigs”) Or Exclude (Exactkeyword, “Guinea Pig”) Or Exclude (Exactkeyword, “Horse”) Or Exclude (Exactkeyword, “Cat Disease”) Or Exclude (Exactkeyword, “Bird”) Or Exclude (Exactkeyword, “Rodentia”).

These terms were selected based on reviews on chemical contaminants in both humans (Wasi et al. 2013; Aryal et al. 2020) and companion animals (Pastorinho and Sousa 2020a). For this study, only the Scopus database was used, considered one of the most important reliable sources of scientific and medical information worldwide (Aria and Cuccurullo 2017; Pranckute 2021), even more than other databases (Harzing and Alakangas 2016; Zhu and Liu 2020). It should be clarified that before choosing only “Scopus,” a preliminary search for the same key terms was performed in other important databases, such as “PubMed” and “Web of Science,” yielding lower results than those found in “Scopus” and with references that were already listed in “Scopus.”

The search was initiated in 1931, the year in which evidence related to this topic was first found in this database. The search ended on September 5, 2022, the date on which the corresponding download was performed (Annex 2). Cited journals, book chapters, and review articles in English were compiled. The data was analyzed with the statistical software R version 4.1.1. The information on authors, keywords, years of publication, *H* index, and institutions among other parameters was extracted by exporting the data in BibTex format from the Scopus database and was analyzed with the Bibliometrix 3.1 package. This package is an open-source tool for quantitative research in scientometrics and bibliometrics that includes all the main bibliometric methods of analysis, such as data matrices for co-citations, couplings, production analysis, scientific collaboration, and joint word analysis (Aria and Cuccurullo 2017).

The information was filtered, corrected, and standardized for the reliability of the results, avoiding bias related to under- or over-representation of the data. Some modifications were necessary, such as double-checking the data, thus avoiding alphabetical biases. Other corrections were made, such as spelling errors, inconsistencies, and the appearance of homonyms in the plain text file downloaded in BibTex format.

## Results

### Documentary production

**Fig. 1:**
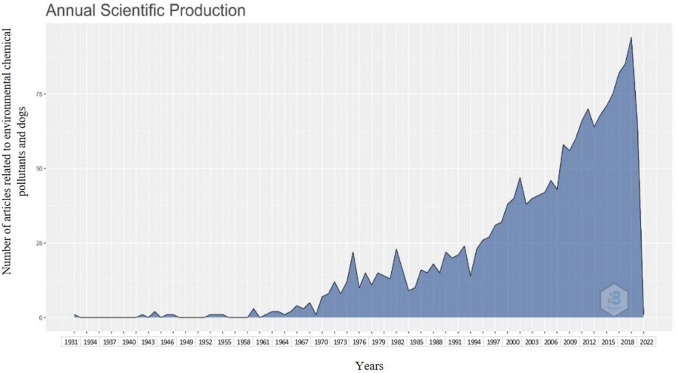
Annual scientific production on research in environmental chemical contaminants and dogs from 1931 to 2022

A total of 1706 documents were extracted from the Scopus database: 1504 articles, 1 book, 53 chapters books, and 148 review articles, that were related to environmental chemical pollutants in dogs. In this analysis, a collaboration index of 4.65 per author was obtained, a value that corresponds to the quotient between the number of authors and the number of articles published (Benavent et al. 2017). An average of 22.67 citations per document and 1878 citations per year per document were obtained. The number of documents fluctuated from one article in 1931 to 65 documents in 2022 (Fig. 1), showing a particular increase in the last decade, being the years 2014 (66 documents) and 2021 (90 documents) the ones with the highest production.

Figure 1 shows that the period analyzed in this review can be divided into four main stages. The first comprised between the years 1931 and 1960 (12 documents), which shows a slight increase in the number of published articles. The second stage is between 1961 and1994 (352 documents), a period when the number of publications remained with an average trend compared to the previous period. The third stage is from 1995 and 2000 (138 documents) a short period in which in less than 5 years half the average number of documents of the previous state were developed. The fourth stage comprised between 2001 and 2022 (1204 documents), a time in which a more rapid development, consolidation, and average annual production were evidenced above the aforementioned years.

### Analysis of publication sources

**Fig. 2:**
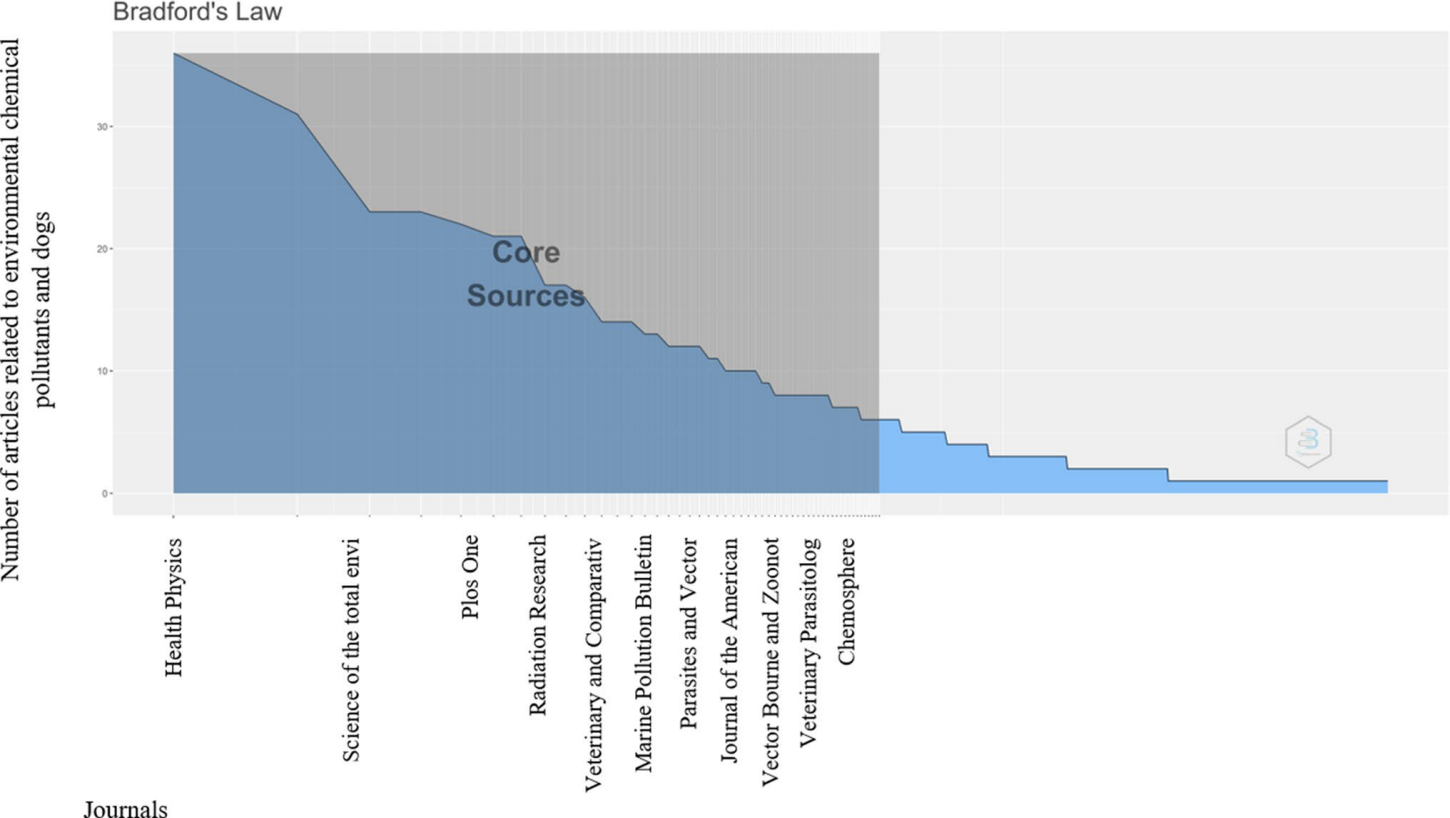
Most relevant publication sources determined according to Bradford’s law of dispersion of the scientific literature in environmental chemical contaminants and dogs

The journals that occupy the first three places are *Health Physics*, *Science of the Total Environment*, and *Plos One* with 36, 31, and 23 articles, respectively (Fig. 2). This was determined by analyzing Bradford’s law of dispersion of the scientific literature and distribution, a law that estimates exponentially decreasing returns from the search for references in scientific journals (Aria and Cuccurullo 2017).

The source local impact evaluated by the *H* index is in a descending order for the journals *Science of the Total Environment*, *Marine Pollution Bulletin*, and *Parasites and Vectors*, having an exponential trend to continue growing according to the trend pattern obtained in the last decade (Fig. 9).

### Author analysis

**Fig. 3:**
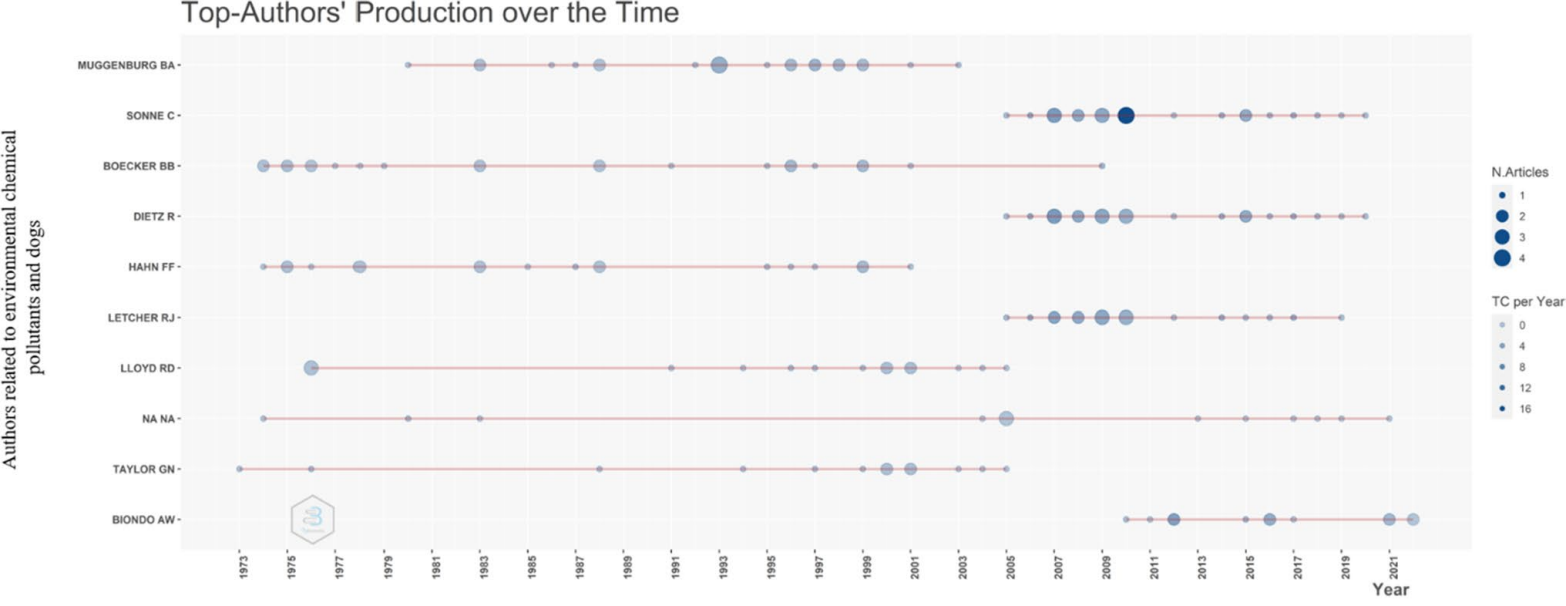
Production of the 10 main authors over time in environmental chemical pollutants and dogs. The straight lines indicate the continuity of studies in relation to time

On the other hand, to determine the impact factor of the authors, the *H* index was taken into account, a system proposed by the University of California that is frequently used to measure the professional quality of scientists and researchers, depending on the number of citations, the articles produced, etc. (Aria and Cuccurullo 2017). The top 10 authors with the greatest scientific production in this field were taken as the top, thus making a relationship between production time and the number of studies that have been developed over time based on the issue of environmental pollutants in dogs (Fig. 3).

For this analysis, Sheinin and Davenport were the authors who initiated the investigations in relation to chemical contaminants in this field in the year 1931. Between 2005 and 2022, Muggenburg B, Sonne C, Dietz R, Letcher R, and Biondo A stand out as the most active authors with an average scientific production of more than five articles produced annually, a very similar average for all five during the years mentioned above (Fig. 3).

**Fig. 4:**
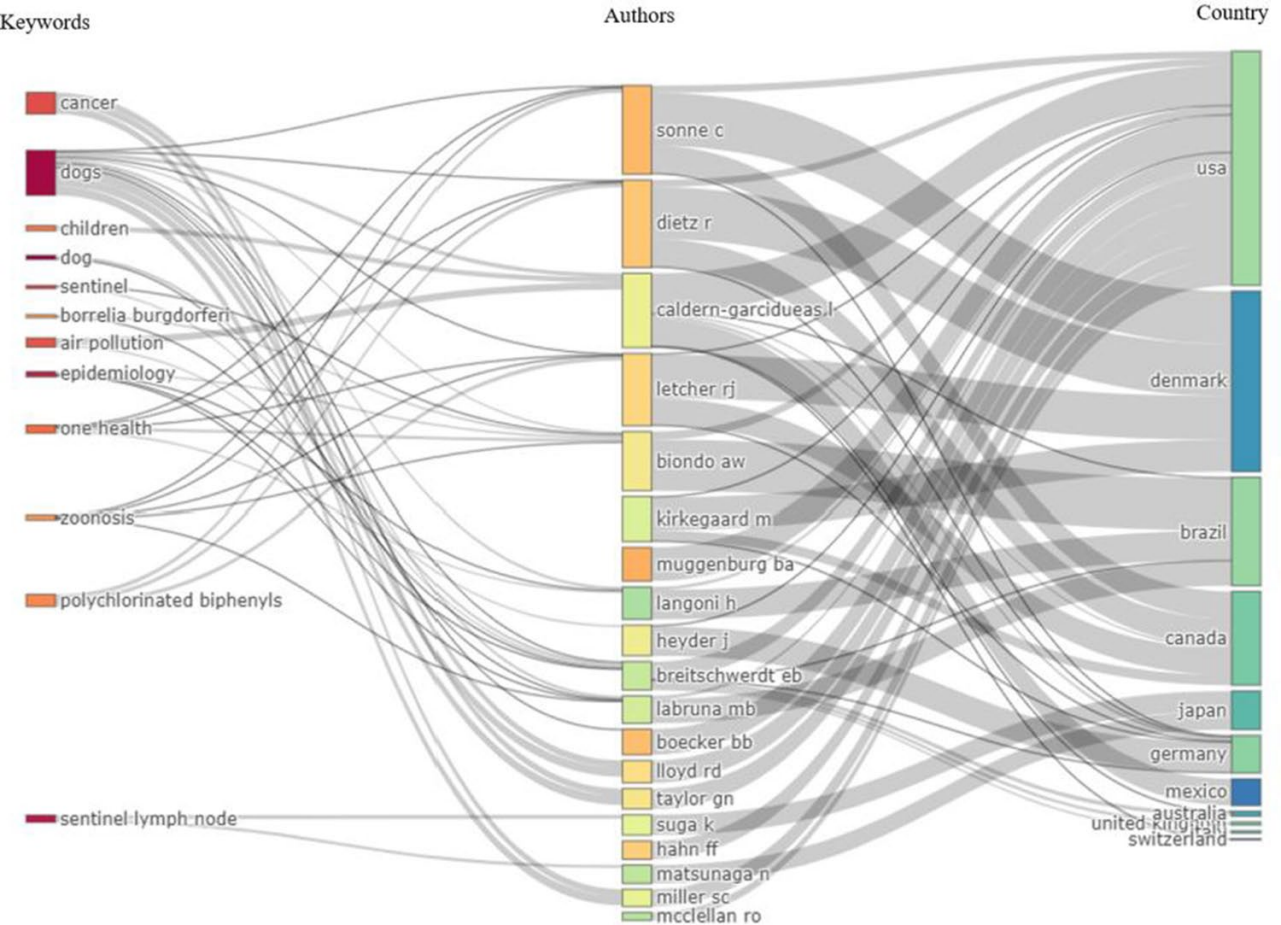
Tripartite graph on publications in environmental chemical pollutants and dogs. Left field, keywords; middle field, authors; right field, countries

Relationships between the keywords, authors, and country of origin were analyzed (Fig. 4). They were graphed in relation to the generation of a top in the following way: the top ten of countries, the top twenty of authors, and the top twelve of the most prominent keywords according to the analysis. Their interconnections and direct relationships that link them were obtained.

Figure 4 shows a clear relationship between the authors Biondo, Sonne, Dietz, and Letcher with keywords such as "dogs", "One Health", "sentinel", and "polychlorinated biphenyls", which suggests the trend of these kinds of studies with the authors and their countries of institutional affiliation respectively. Likewise, it is observed that authors such as Heyder, Kirkegaard, and Breitschwerdt have had a prominent role in these issues with affiliations to eastern countries such as Japan and China, this being convenient when managing to broaden the spectrum of this type of research line in Asian countries. The keywords with the highest number of repetitions cited by the authors were "sentinel", "air pollution", and "dogs".

### Institutional affiliation analysis

**Fig. 5:**
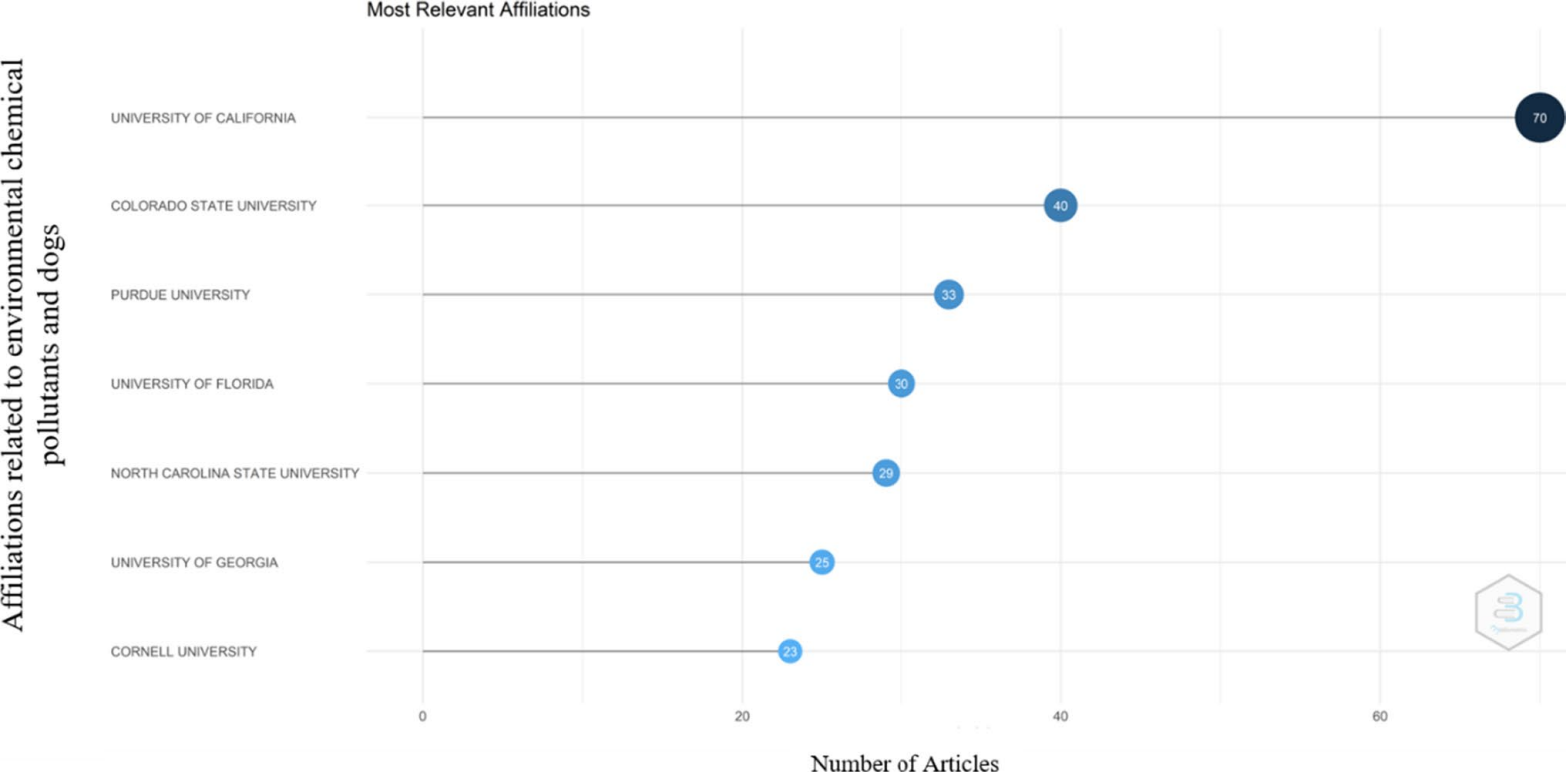
Most important affiliations for the development of studies in environmental chemical contaminants and dogs

The results showed that the University of California (USA) has a total of 70 publications, while the University of Colorado State (USA) and the University of Purdue (USA) showed a total of 40 and 33 publications each (Fig. 5). However, other leading universities in the world such as the University of Florida and the University of North Carolina also appeared as affiliations interested in approaching this type of research.

**Fig. 6:**
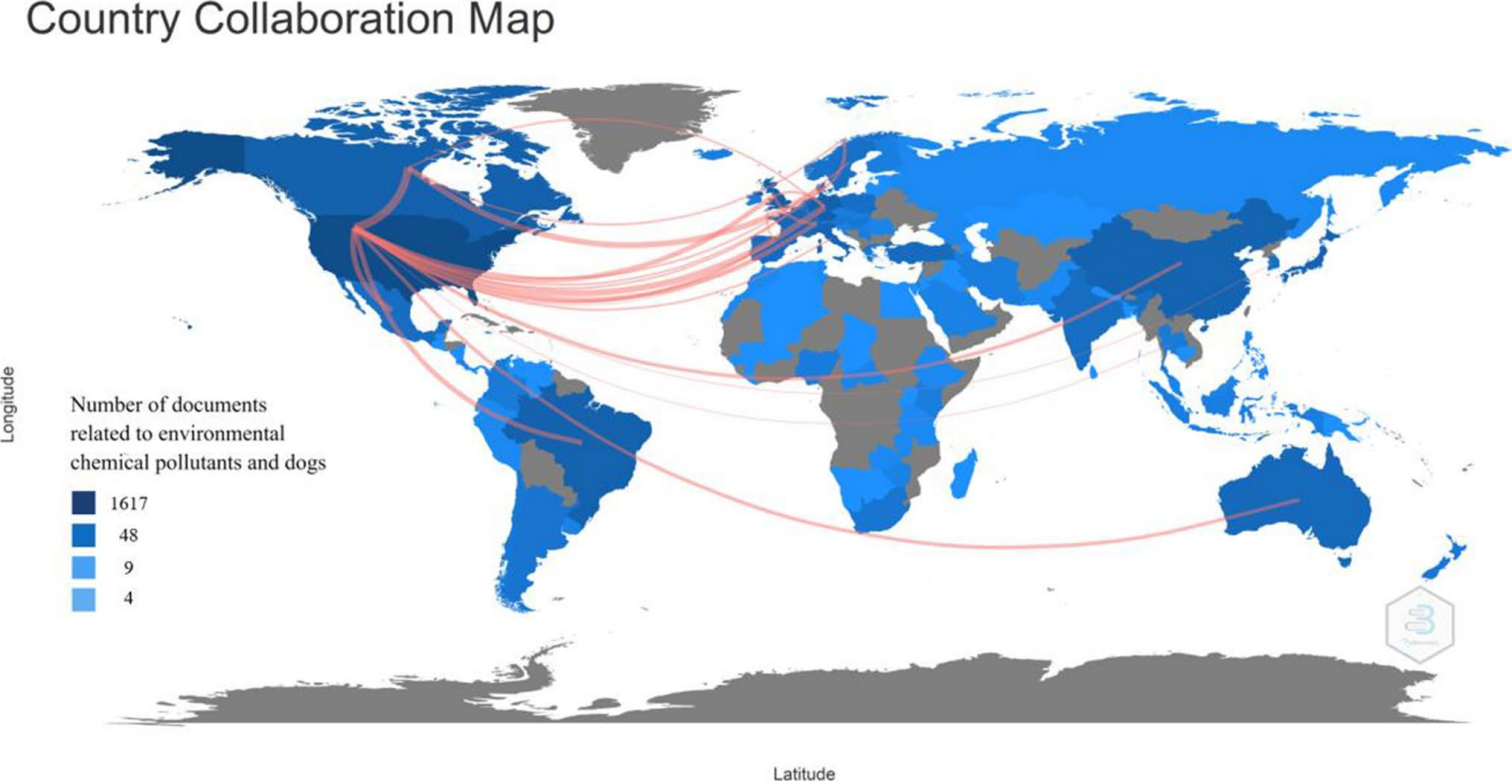
Map of production and collaboration between countries in environmental chemical pollutants and dogs. The edges indicate the networks of nodes that exist between countries for the development of this type of research; the thicker lines indicate the existence of a stronger collaboration between countries. The conventions on the left side indicate the average number of documents produced for each country

The countries with the highest production are the USA, Brazil, Germany, and the UK with a total of 1545, 283, 228, and 197 scientific productions, respectively. This number corresponds both to each country’s own authorship and to its collaboration networks. Higher collaborations were observed between the USA and Canada (35 edges) and Germany (21 edges). Collaborations between countries such as the UK with Switzerland (6 edges) and France (6 edges) were also observed (Fig. 6). For South America, Brazil has a higher rate of collaboration with the UK (3 edges). Therefore, the countries of the Northern hemisphere are the ones with a higher number of collaboration networks and therefore are the most cited for the development and consolidation of this type of research.

### Network analysis

#### Keyword analysis

**Fig. 7:**
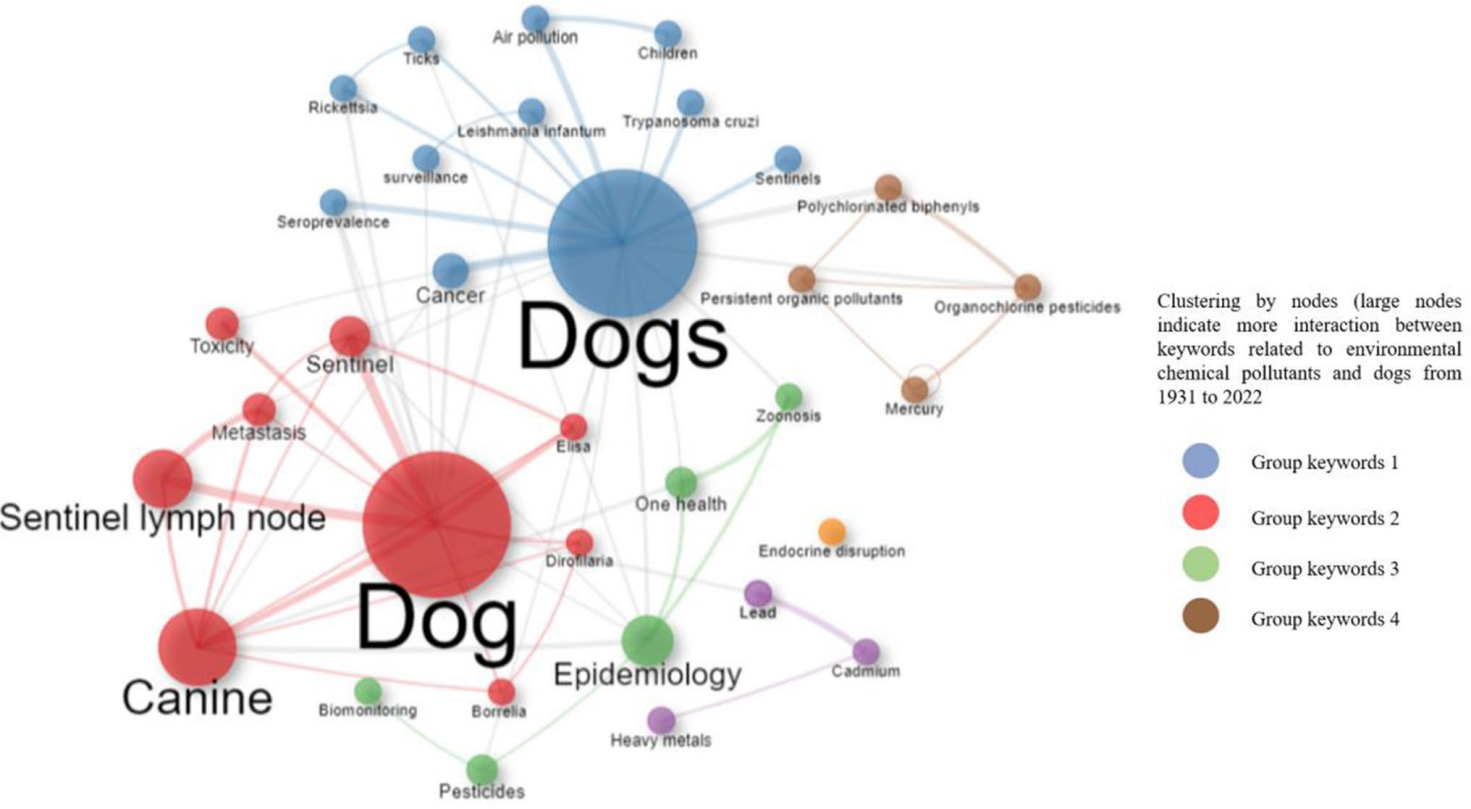
Association networks between the words most used by the authors between 1931 and 2022 in environmental chemical pollutants and dogs. The colors indicate the association between these words

A keyword analysis is a fundamental tool to identify the most relevant terms in research development (Harzing and Alakangas 2016; Zhu and Liu 2020). In this sense, the Biblioshiny interface generated a list of top 30 words according to their frequency of use in the articles analyzed, which required a series of transformations. First, Louvain’s grouping algorithm was applied to this number of words, which performs and guarantees an evaluation of the data set, comparing the density of edges that are present inside or outside the community (Aria and Cuccurullo 2017). Next, the results were cleaned up in order to eliminate synonymous words and terms not associated with the topic (Fig. 7).

It was possible to identify four groups with the highest representation of frequencies for the keywords (Fig. 7). The largest group, represented in blue, focuses on "sentinels", "air pollution", "children", and "cancer". The next group, represented in red, includes words related to the specific effects on the species, such as "toxicity", "sentinel", and "metastasis", and their clear relationship with dogs and the effects of this pollutant in this species. The third group is the smallest and is represented in green, focusing its research on "epidemiology", "biomonitoring", "One Health", and "pesticides". The fourth group, one of the smallest and is represented in brown, focuses its research on "persistent organic pollutants", "polychlorinated biphenyls", "organochlorine pesticides", and "mercury".

The words with the highest concurrence, that is, with the highest node size that were identified in the graphic interface were the following: "dogs", "canine", "sentinel lymph node", and "epidemiology". Similarly, but not less important, within the nodes with lower frequency of occurrence were found the words "cadmium", "lead", "endocrine disruption", and "heavy metals". Finally, terms such as "canine diseases", "risk assessment", "risk factors", and "veterinary medicine" have been detected in this analysis as trending topics during the last 5 years (Fig. 10).

#### Co-authorship analysis

**Fig. 8:**
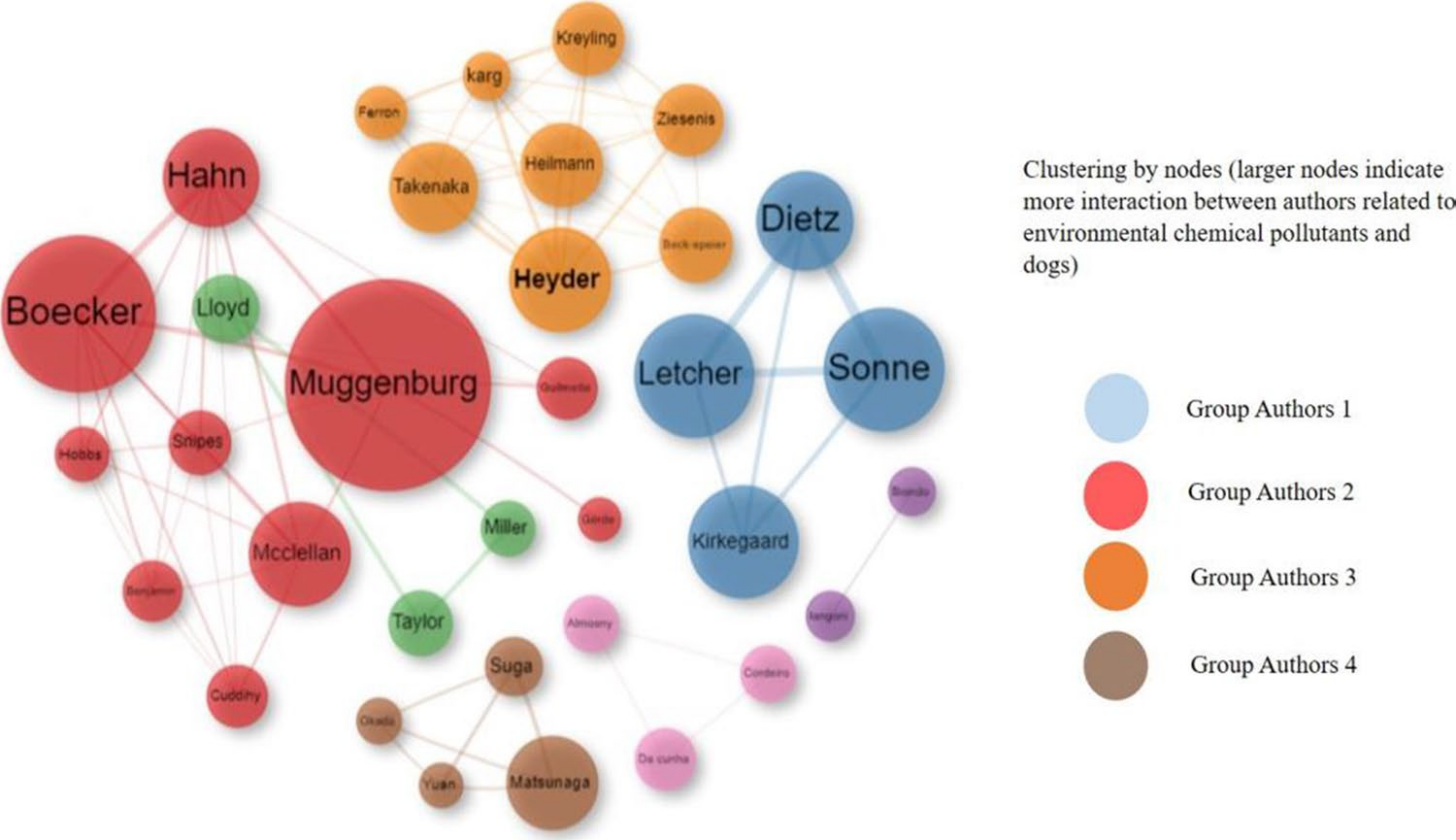
Citation networks in environmental chemical pollutants and dogs between 1931 and 2022

**Fig. 9:**
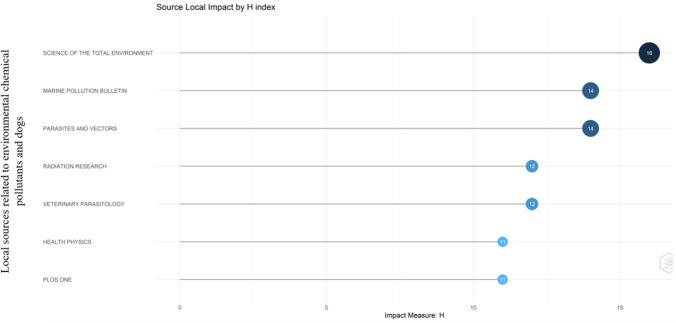
Top four most cited local sources

**Fig. 10:**
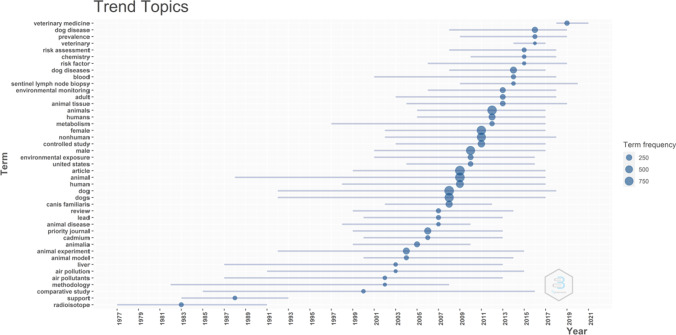
top trending topics by frequency term from 1931 to 2022

The top authors and the key topics of interest were analyzed. The inclusion criteria for this review were the top 50 of the most cited authors using a series of criteria applied by the interface. For example, the selected authors were those who participated in at least five publications. Each node used corresponds to an author, while the ratio of node sizes represents the number of publications generated. Subsequently, and prior to the filtering adjustments and spelling corrections, Louvain’s algorithm was used for the grouping of the data obtained, finding an estimate of the grouping of nodes represented in Fig. 8.

The results of this graph showed the most representative authors were Sonne, Dietz, Letcher, Kirkegaard (grouping in blue), Boecker, Muggenburg, and Hahn (grouping in red) and Heyder, Takenaka, and Heilmann, (grouping in yellow) being the most prominent authors of each group. In this sense and as seen in the tripartite graph, Sonne, Muggenburg, and Heyder continue to be the most representative nodes, because they are the authors with the highest frequency of citation for this analysis. Likewise, the existence of a series of networks clearly shows a close collaboration between their studies.

## Discussion

### Knowledge gaps

A bibliometric analysis was carried out on chemical environmental pollutants and dogs for a better understanding of collaboration networks and research trends. To the knowledge of the authors, this is the first bibliometric analysis where dogs are considered a study model for the evaluation of chemical contaminants worldwide.

This study showed a relatively lower amount of bibliographic material (1706 documents) in comparison to similar fields of research related to human health risks, such as air pollution and human health (2179 documents; Dhital and Rupakheti 2019), or climate change and human health (4247 documents; Sweileh 2020). There is also a relatively lower amount of bibliographic material in dogs in comparison to other animals such as fish (38,739 documents), rats (21,995 documents), birds (8233 documents), pigs (4537 documents), and rabbits (2715 documents), when the same search was carried out in the Scopus database, but for other animals. This could be related to the fact that for many years, the development of different disciplines such as veterinary sciences, human health, and ecotoxicology was done separately and that usually, studies in dogs were carried out exclusively in the field of veterinary sciences. However, most of these animals exhibit more important differences in comparison to humans, in physiology and metabolism, than dogs. Therefore, the effects of chemical contaminants in these animal models are difficult to extrapolate to humans (National Research Council (NRC) 1991; Parker et al. 2006; Hoffman et al. 2018).

It is interesting to notice that the amount of bibliographic material in dogs is higher in comparison to other pets, such as cats (322 documents). This could be explained by the fact that dogs and humans have more similar enzymatic and metabolic systems, than cats and humans (Dalgaard 2015). In addition, humans and dogs respond to many toxic substances in an analogous way (Marcella et al. 2006). Research on environmental chemical contaminants and dogs has grown considerably in the last 5 years, both in the collaboration rate and in the number of publications. This could be related to an increase in collaborative efforts of multiple disciplines such as those proposed by One Health, where ecologists, veterinarians, and medical doctors play a considerable role (WHO 2020; Humboldt et al. 2020). For example, new studies on zoonosis and One Health are using dogs as sentinels (Bowser and Anderson 2018; Sonne et al. 2020), and studies on canine diseases, risk assessment, risk factors, and veterinary medicine are becoming trending topics (Hathaway 1991; Environmental Working Group (EWG) 2008; Hoffman et al. 2018; Pinello et al. 2022). The increase in scientific production could also be related to similar results found recently in dogs and humans (Chen et al. 2022), such as an increased micronucleus frequency in dogs (Harper et al. 2007) exposed to pollutants (Backer et al. 2001), also found in humans exposed to pollutants (NRC 1991; O´Callaghan-Gordo et al. 2015; González et al. 2021).

Countries with higher scientific production are mainly located in the Northern hemisphere. Eastern countries such as Japan and China are recently playing an important role in research areas such as air pollution, particulate matter pollution, and dogs as sentinels of these types of pollution (Zhang et al. 2017; Chen et al. 2022). However, even if dogs have been used as sentinels for tropical diseases, diseases related to air pollutants (Calderon et al. 2001), and aspects related to One Health (do Couto et al. 2022), the development of more research studies in other regions of the world, such as Latin America, is still missing, especially in areas with constant emissions of chemical pollutants (i.e., Hg) (Canham et al. 2021; UNODC 2020).

In addition, despite a considerable increase in scientific production, there are important knowledge gaps that need to be addressed such as the development of biomarkers of exposure and toxic effects that reflect similar biological events in both humans and dogs and studies associated with dogs and heavy metals, pesticides, and endocrine disruptors. Other important subjects need to be addressed in future studies, such as environmental differences in different regions, spatial and temporal distribution of contaminants, most common routes of exposure, and their mapping, as well as racial and metabolomic differences in dogs, among others. In addition, drastic differences exist in the metabolism and biomagnification of different pollutant metabolites among humans and dogs, which is why more studies are needed in this research field (NRC 1991; Pastorinho and Sousa 2020a, b).

### Topics of lasting impacts

The most cited references described in this document highlight the relevance of the work of each research. It should be emphasized the influential role of Sonne (2010), Sonne et al. (2011, 2014a, b, 2015) and Letcher et al. (2009, 2017), who focus their studies specifically on the effect and relationships that have dogs exposed to different emerging environmental pollutants. For example, Lau et al. (2017) reported in dogs the existence of persistent pollutants such as *PBDEs*, and *PCBs* (660 and 1371 ng/g in lipids), chemicals that tend to accumulate on the marine trophic chain, finding high concentrations of these compounds in arctic and circumpolar areas (Sonne et al. 2020). It would therefore be feasible for sled dogs to be considered sentinel animal species suitable for monitoring the health of particular ecosystems. Moreover, in a brief recapitulation of studies, heavy metals such as Hg (0.1–3 ppm hair), lead (Pb) (0.2–2.5 ppm hair), and cadmium (Cd) (0.02–2.5. ppm hair) were pollutants with high emissions in urban centers and mining areas and were detected at high concentrations in dogs (Toyomaki et al. 2020; Pastorinho and Sousa 2020a). Therefore, dogs could be used as sentinels for their effects on human populations, using noninvasive matrices such as hair (Pastorinho and Sousa 2020a). In Asia, research conducted by Zhang et al., Wang et al., and Chen et al. has addressed important issues such as water quality, biological pollutants, and air pollution, among others (Wang et al. 2010, 2020a, b; Zhang et al. 2019; Chen et al. 2021). In this sense, the use of potential indicator species that present a potential link between the environment, wildlife, and humans is required, being the dog as one of the animals that have most shared diseases with humans, generating synanthropic life cycles, i.e., systems specialized for the relationship of diseases between man and dog.

One of the clearest limitations of the study is the small number of studies currently available, which, although relatively low compared to other disciplines, shows a clear upward pattern. In addition, contaminants have a spatial distribution that depends on the socioeconomic activities of the region. Therefore, there are probably other regions of the world to pay special attention to, especially in countries with little environmental regulations, in which dogs could be very useful as sentinels of environmental chemical contamination, for example, mining areas that have been negatively impacted by the development of this type of activity in Africa (Toyomaki et al. 2020) or Latin American countries (UNODC 2020; Carriquiriborde 2021) where sample collection proves to be somewhat problematic.

It is important to clarify also that this search was only done in English, which excludes local studies published in other languages, such as Spanish. For example, German and Portuguese languages have been gaining strength recently, with 53 and 33 publications, correspondingly, in the last year. For this reason, a call is made to develop more bibliographic and review articles that complement this study, since this is, to our knowledge, the first bibliometric analysis carried out in this field.

## Conclusion

An exponential increase in scientific production in dogs and environmental chemical contaminants has been recorded in the last 90 years, especially in the last 5 years. This should probably continue to increase in the upcoming years. More multidisciplinary and interdisciplinary research studies have been carried out in the Northern hemisphere. There is a growing interest in the development of various lines of research related to persistent organic compounds (PCBs, PBDEs, pesticides) and heavy metals (Hg and lead mainly), using dogs as sentinels, as well as new lines of research related to zoonosis and One Health. Additionally, issues related to pollutants, sentinel lymph nodes, and epidemiology appear also as new lines of research. However, major collaborations with countries in the Southern hemisphere are needed for worldwide and local studies in different regions of the world. We also recommend the integration of new approaches such as the development of biomarkers of exposure and toxic effects that reflect similar biological events in both humans and dogs. New findings from investigations on the effects of environmental pollution on dogs could be very relevant to human health, lead to public health interventions and policy initiatives, and considerably help to provide an early, useful, and urgently needed warning system for public health intervention.

## Data Availability

Two files related to the top four most cited local sources are attached, and another file in relation to the Scopus database that was used in Bibliometrix of R for the execution of bibliometric analyses. In addition, the data that support the findings of this study are available from the corresponding author upon request.

## Appendix

**Annex 2 **Data downloaded from Scopus on the keywords used for your search (see PDF file)

See Figs. 9, ^10^

## Declarations

### Disclaimer

The publisher will not be legally liable for compensation claims.
